# Persistent effects of experimental heatwave duration on population dynamics

**DOI:** 10.1098/rsbl.2026.0444

**Published:** 2026-10-21

**Authors:** Emma B. Gruntz, Tess N. Grainger

**Affiliations:** Department of Integrative Biology, University of Guelph, Guelph, Canada

**Keywords:** heatwave, climate change, temperature, extreme weather events, population dynamics, mortality, fecundity, *Tribolium*

## Abstract

Heatwaves are becoming longer, more frequent and more intense under climate change. However, we still have a limited understanding of the longer term (multi-generational) impacts of heatwaves, and of the demographic mechanisms that underly these effects. To address these gaps, we conducted an experiment using a model system of flour beetles (*Tribolium castaneum*) that tested the effects of heatwave duration on population dynamics. We subjected replicate beetle populations to a single 38°C heatwave event of one of four durations (0, 5, 10 or 15 days) and then tracked population sizes, birth rates and mortality rates for 7 months (approx. 7 generations). We found that, initially, heatwaves reduced population growth rates by temporarily lowering birth rates (via reduced fecundity), but that later in the experiment, the impact of heatwaves on population dynamics occurred via increased mortality rates. Together, these demographic effects resulted in a sustained suppression of population sizes throughout the experiment, as populations exposed to longer heatwaves (10-day and 15-day) experienced reduced carrying capacities for the duration of the experiment. Our findings indicate that heatwaves can have persistent negative impacts on population dynamics that could be underestimated by focusing solely on short-term responses.

## 1. Introduction

Climate change is causing heatwaves to become longer, more frequent and more intense [1–3]. Heatwaves, broadly defined as a period of 5 or more days in which temperatures exceed some threshold above the norm [4–6], have been shown to affect a wide range of biological processes, from survival and fertility to behaviour and species interactions [3,7–9]. Indeed, the devastating impacts of heatwaves on wild populations have received increased attention in recent years [10–13], and in many systems extreme temperature events have a stronger impact on ecological communities than increasing average temperatures [8,11]. However, in the face of accelerating climate change, there is a pressing need not only to document how heatwaves are impacting natural communities, but also to clarify which types of heatwaves cause the most detrimental impacts, and which mechanisms drive these effects [8]. Determining how attributes of heatwaves such as their timing, frequency and duration drive biological responses, and identifying the traits and demographic responses that underly these effects, will be necessary to predict population stability and the risk of extinction under global change. Answering these questions requires controlled heatwave experiments [14].

Experiments have provided critical insights into the effects of various aspects of heatwaves on biological systems, including their timing [15–17], severity [18–20] and frequency [20,21]. However, experiments with longer heatwave durations that cover a substantial portion of an organism's life cycle and that are relevant to projected climate change conditions (i.e. weeks or months) remain less common (but see [22,23]). Perhaps more critically, heatwave experiments have primarily focused on short-term responses at the individual scale, which leaves us with a lack of understanding of the longer term population-level impacts of heatwaves that manifest or endure across multiple generations [3,9,24,25]. As such, it remains unclear whether short-term effects can predict longer term responses, whether populations tend to recover from heatwaves over time, or whether negative impacts that are not immediately apparent could manifest in subsequent generations [13,24,25].

To fill these gaps, we tested the effects of heatwave duration on population dynamics by exposing populations of red flour beetles (*Tribolium castaneum*) to a single 38°C heatwave event lasting for one of four durations: a no-heatwave control, 5, 10 or 15 days. We then returned all populations to optimal conditions (30°C) for 7 months (approx. 7 generations) and tracked population sizes, mortality rates and birth rates through time. We also measured the effect of heatwaves on fecundity at three timepoints (0, 1 and 3 months post-heatwave), and on body size and sex ratios once (3 months post-heatwave). Overall, we found a lasting effect of longer heatwaves (10-day and 15-day) on population dynamics, indicating that the duration of a heatwave is a critical determinant of the severity of its ecological effects, and that the effects of heatwaves can persist for generations.

## 2. Material and methods

### (a) Study species

For our experiment, we used the red flour beetle (*T. castaneum*), a global pest of stored grain products that has served as a model system in ecology and evolutionary biology for over a century [26]. In particular, the response of *T. castaneum* to high temperatures is well resolved owing to its use in heatwave experiments [16,27,28], and the use of high temperatures to eradicate it from stored grain [29,30]. The optimal temperature for fecundity and population growth for *T. castaneum* is 30–32°C, with development from eggs to larvae to pupae to adult occurring in approximately 30 days at these temperatures [31–34]. Previous heatwave experiments with this species indicate that 5 or more days at 5–7°C above the strain's optimal temperature negatively affects this species’ reproduction and survival, but is not completely lethal [16,28]. Flour beetle life history is marked by frequent dispersal, colonization and population expansion as they move between grain storage facilities [26,35]. Mated *T. castaneum* females lay approximately 1–10 eggs per day, females produce eggs continuously throughout their lives, and adult lifespan is approximately 6 months [31,36]. This species also experiences cannibalism in which larvae primarily consume eggs and adults consume eggs, larvae and pupae [26,37]. The beetles used in this experiment were a laboratory stock (‘COL-2’) from the United States Department of Agriculture (USDA) that has been maintained on whole wheat flour at 30°C for approximately 35 years. Since their acquisition from the USDA in 2021, stock populations have been maintained by serial transfer on whole wheat flour and kept in Panasonic PCHbi incubators at 30°C, total darkness and 60 ± 10% humidity.

### (b) Experimental design

To determine the effect of heatwave duration on flour beetle population dynamics, we conducted an experiment with four treatments: a control (no heatwave), or a 5-, 10- or 15-day heatwave. For each of our four treatments, we had 10 independent replicates (beetle populations), for a total of 40 populations in the experiment. During heatwaves, beetles were kept at 38°C, and during all non-heatwave heatwave periods (before and after heatwaves), beetles were kept at 30°C, and humidity was always 60 ± 10%. We selected 5 days as our shortest heatwave based on the commonly used minimum duration of 5 days for an extreme heat event to classify as a heatwave [5,6]. We set our range of durations (5–15 days) to be reflective of summer heatwave durations in recent years across Canada [38]. We set our heatwave intensity at 38°C because an 8°C increase in temperature (above this strain's historic temperature of 30°C) reflects increases above the normal daily high that have been recorded in recent heatwave conditions in Canada, and because previous heatwave experiments with *T. castaneum* have also used 5 or more days at 5–7°C above the strain's optimal temperature [16,28]. We note that our experimental heatwaves are probably a conservative estimate of the impacts of heatwaves on this species, as natural heatwaves can last much longer than 15 days [39,40], and because temperatures exceeding 38°C are common in many countries inhabited by our focal species [16,41].

To initiate each experimental population, we placed 55 randomly selected unsexed *T. castaneum* adults into a plastic container (6 × 4 × 4 cm) containing 25 g of organic whole wheat flour, and randomly assigned each population to one of four heatwave duration treatments. At the start of the experiment, eight populations suffered an experimental mishap that resulted in escaped beetles, and these populations were discarded, leaving seven to nine replicates per treatment in the experiment (control *n* = 8; 5-day *n* = 7; 10-day *n* = 9; 15-day *n* = 8). All populations were placed in an incubator (Panasonic PCHbi) set to 30°C, 60 ± 10% humidity, and total darkness and left for 10 days. We allowed this 10-day gap between starting our populations and starting our heatwaves in order to create a biologically realistic heatwave scenario in which multiple life stages were exposed to heatwaves. *T. castaneum* adults mate and lay eggs continuously, and at 30°C their life cycle is completed in approximately 30 days with an egg stage of approximately 3–4 days, a larval stage of approximately 20 days and a pupal stage of approximately 4–5 days [26]. As such, the 10-day gap meant that when the heatwaves began, each population contained a mixture of the originally added adults and their offspring (eggs and larvae).

After this initial 10-day period, we initiated heatwave treatments by transferring populations within each treatment into another incubator set to 38°C and the same humidity and light conditions described above. We staggered the start of our heatwave treatments by starting the 15-day heatwave first, then 5 days later we started the 10-day heatwave, and then 5 days after that we started the 5-day heatwave. We did this so that all heatwaves would end on the same day, allowing us to measure our responses simultaneously across all treatments. Thus, when the heatwaves started, all populations contained adults, eggs and early-stage larvae; the 10-day heatwave populations additionally contained some mid-stage larvae, and 5-day heatwave populations additionally contained some later-stage larvae. We kept the control populations at 30°C for the duration of the experiment. At the end of the heatwave period, populations from all treatments were moved into a 30°C incubator and kept there for 7 months until the end of the experiment.

### (c) Population dynamics

#### (i) Population size

To assess the effect of heatwave duration on population dynamics, we continued to maintain all populations (at 30°C) and to monitor populations for 7 months after the conclusion of the heatwaves. Experiments with flour beetles either impose discrete generations (by removing adults at the start of each new generation) (e.g. [42]), or allow beetles to experience continuous overlapping generations (e.g. [43,44]). Here, we allowed populations to have continuous overlapping generations (adults were never removed) in order to mimic the natural life history of this species and allow beetles exposed to the heatwave to remain in the experiment and experience any delayed effects. We refer to the 7-month duration of our experiment as seven generations because it takes *T. castaneum* approximately 1 month to undergo one full generation (egg to adult) [26,34].

We counted the number of live adult beetles and dead adult beetles the day that the heatwaves ended (‘month 0’) and then again 1 month (4 weeks), 3 months, 5 months and 7 months after the heatwaves ended. To do this, we separated beetles from their flour habitat using a sieve (no. 25, 710 m, Kimlab) and then manually counted all adult beetles. Each month we replenished the beetles’ resources by replacing half of the flour in each population's container with fresh flour. Although some eggs and small larvae are lost each month in the discarded flour, these are rapidly replaced via continuous egg laying, and this does not affect adult population sizes (figure 1A). After each population count and/or resource replenishment, we returned all populations to the 30°C incubator.

**Figure 1.**
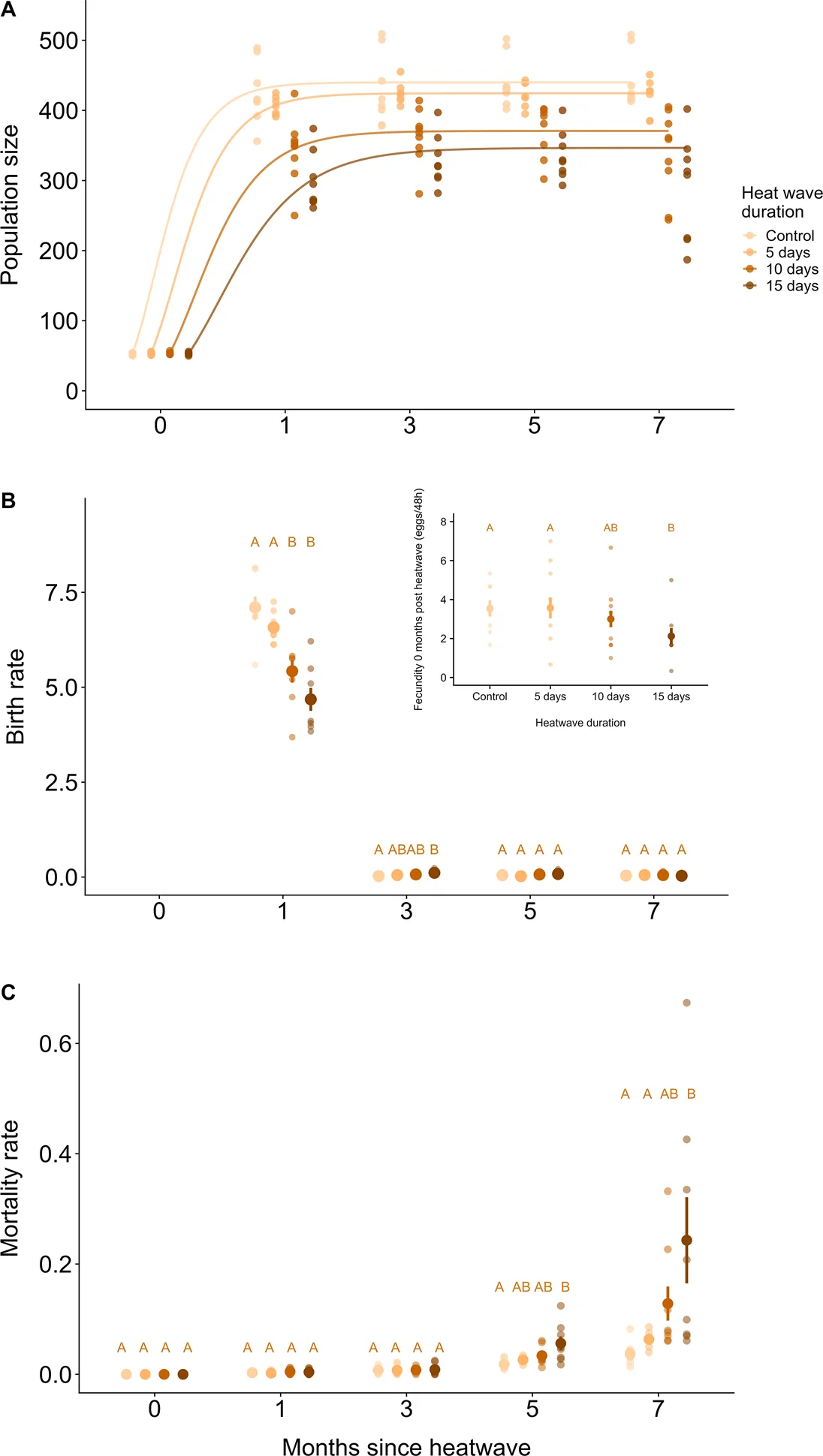
The effect of heatwave duration on *T. castaneum* (A) population dynamics, (B) birth rates and (C) mortality rates following heatwaves of 0 (control), 5, 10 or 15 days. Heatwaves were all set at a constant 38°C, whereas the control treatment experienced a constant 30°C and no heatwave. Counts taken at 0 month since heatwaves were conducted the day the heatwaves concluded. In (A), population size is the number of live adult beetles, and lines show predictions from the fitted Gompertz population growth model for each treatment. In (B,C), solid points represent treatment means, faded points represent individual replicate populations, error bars show the s.e.m., and letters above points indicate significant differences across treatments (*p* < 0.05). Full results are shown in table 1.

#### (ii) Birth rate and mortality rate

We calculated birth rates and mortality rates at each survey date (0, 1, 3, 5, 7 months post-heatwaves). We defined birth rate as the number of new adult beetles in the population at each count divided by the population size at the previous count, according to the following formula:

(2.1) $$\begin{array}{r} birth\;rate\;=\frac{n_{t}+d_{t}-n_{t-1}}{n_{t-1}}, \end{array}$$

where $n_{t}$ is the number of live adult beetles at the current timepoint, $d_{t}$ is the number of dead adult beetles at the current timepoint and $n_{t-1}$ is the number of live adult beetles at the previous timepoint. In cases where the number of births was negative (indicating a minor counting error), the birth rate was set to zero. We calculated mortality rate as the number of dead adult beetles divided by the number of live adult beetles at each timepoint.

### (d) Traits

#### (i) Fecundity

We assessed the effect of heatwave duration on fecundity (egg production) at three timepoints: immediately after the heatwaves ended (0 month), and at 1 and 3 months after the heatwaves. To do this, at each of these timepoints, we randomly selected and removed three female adult beetles from each population and placed each female in an individual vial containing 0.8 g whole-wheat flour. We allowed these individuals to lay eggs for 48 hours and then sifted eggs out of the flour, placed them under a microscope to be counted, and disposed of the females.

#### (ii) Body size

We assessed the effect of heatwave duration on adult body size 3 months after the heatwaves. To do this, we collected five female and five male adult beetles from each population, dried them at 60°C for 48 hours, and then weighed beetles individually.

#### (iii) Sex ratio

We assessed the effect of heatwave duration on sex ratios 3 months after the heatwaves. To do this, we randomly selected 10 adult beetles from each population, recorded their sex and then returned them back into their respective populations.

#### (iv) Data analysis

To determine the effect of heatwave duration on population dynamics (intrinsic growth rate and carrying capacity), we fitted a Gompertz population growth model for each population. On a log scale, the Gompertz model is a simple linear difference equation $x_{t}=\;a+bx_{t-1}$, where $x_{t}$ is the natural log of population size at time *t*, a is the population's intrinsic growth rate, b is the strength of intraspecific competition, and the natural log of carrying capacity can be calculated as $log(K)\;=\;x_{\infty }=\;a/(1-b)$ [45] (see supplementary material for full description of the model). Then, to test the effect of heatwave duration on a and K, we conducted a random-effects meta-regression using the *metafor* package, with heatwave duration as a categorical moderator, the estimated growth rate (a) or carrying capacity (K) of each population as the response variable, and between-replicate heterogeneity estimated using restricted maximum likelihood (REML), such that each replicate's weight in the model reflected its sampling precision and the estimated heterogeneity among replicates. We assessed the effect of heatwave duration on a and K by comparing the full model with a reduced model lacking the treatment moderator using likelihood ratio tests. When a treatment effect was significant, we used Tukey-adjusted pairwise comparisons (with the *emmeans* package) to determine which treatment levels differed from one another.

To determine the effect of heatwave duration on birth rates and mortality rates at each survey date, we used linear models with heatwave duration as a fixed effect and the natural log of either birth rate or mortality rate as the response variable. We checked model assumptions visually using qq plots and plots of residual versus fitted values, and used the natural log (with an added constant of 0.001) of birth and mortality rate as our response variables in order to meet the assumption of normality. We then ran an ANOVA on each model, followed by *post hoc* Tukey tests.

To determine the effect of heatwave duration on fecundity, we used a generalized linear mixed effects model at each timepoint at which fecundity was measured (0, 1 and 3 months post-heatwave). Each model had heatwave duration as a fixed effect, population replicate as a random effect (to account for the lack of independence of the three females measured per population), and fecundity (number of eggs laid by each female in 48 hours) as the response variable. We checked model assumptions with diagnostic tests using the *dHARMA* package. In cases where there was over-dispersion and zero-inflation (months 1 and 3), we used a zero-inflated negative binomial error distribution (with the *glmmTMB* package), and in cases where there was not (month 0), we used a Poisson error distribution (with the *lme4* package).

To determine the effect of heatwave duration on body size and sex ratio (each measured at 3 months post-heatwave), we used linear models with heatwave duration as a fixed effect and either and body size (average dry weight of five individual beetles per population) or the natural log of sex ratio (proportion females out of 10 randomly selected individuals) as the response variable. We checked model assumptions visually using qq plots and plots of residual versus fitted values, and conducted a one-way ANOVA on each model.

All data analyses were conducted in R (version 4.5.0), all figures were made using the *ggplot2* package, and all data and code are available on Zenodo at https://doi.org/10.5281/zenodo.21705081 [46].

## 3. Results and discussion

In this study, we assessed the effects of heatwave duration on population dynamics using an experimental system of *Tribolium* flour beetles. We found that despite a lack of any immediate heatwave-induced mortality, the persistent negative effects of our heatwaves became apparent as the experiment progressed. Specifically, our heatwaves reduced population growth rates (*χ*^2^ = 20.76, d.f. = 3, *p* < 0.001) and then caused a reduction in carrying capacity that was both substantial (up to approx. 25%) and sustained (7 generation-long) (*χ*^2^ = 31.86, d.f. = 3, *p* < 0.001) (figure 1A). We also found that longer heatwaves were required to elicit a response, as only 10- and 15-day heatwaves had significant negative effects on the responses we measured (table 1, figure 1). This finding of a sustained suppression of carrying capacity by our longer heatwaves warrants attention, as it contrasts with the dominant understanding of heatwave responses as characterized by initial detrimental effects followed by a period of recovery [24]. Although heatwaves that cause immediate high levels of mortality are well documented, our study demonstrates the importance of more subtle and longer term impacts of sub-lethal heatwaves on population dynamics [18,25].

**Table 1.** The effect of heatwave duration on *T. castaneum* intrinsic growth rates, carrying capacity, birth rate and mortality rate (each measured at five timepoints), fecundity (measured at three timepoints), and body size and sex ratios (each measured at one timepoint).

**intrinsic growth rate** (a)	**significant contrasts**	**d.f.**	***χ*^2^**	***p***	**figure**
	control–10 d control–15 d 5–15 d	3	20.76	**<0.001**	1A
**carrying capacity (**K)	**significant contrasts**	**d.f.**	***χ*^2^**	***p***	
	control–10 d control–15 d 5–10 d 5–15 d	3	31.86	**<0.001**	1A
**birth rate**	**months since heatwave**	**d.f.**	***F***	***p***	1B
	0 mo	NA	NA	NA	
	1 mo	3–28	14.23	**<0.001**	
	3 mo	3–28	3.66	**0.02**	
	5 mo	3–27	1.14	0.35	
	7 mo	3–27	1.25	0.31	
**mortality rate**	**months since heatwave**	**d.f.**	***F***	***p***	1C
	0 mo	NA	NA	NA	
	1 mo	3–28	0.46	0.72	
	3 mo	3–28	0.14	0.94	
	5 mo	3–27	6.08	**0.003**	
	7 mo	3–28	9.59	**<0.001**	
**fecundity**	**months since heatwave**	**d.f.**	***χ*^2^**	***p***	
	0 mo	3	10.23	**0.017**	1B
	1 mo	3	2.24	0.52	S1
	3 mo	3	3.82	0.28	
**body size**	**months since heatwave**	**d.f.**	***F***	***p***	S2
	3 mo, females	3–28	0.89	0.46	
	3 mo, males	3–28	0.16	0.92	
**sex ratios**	**months since heatwave**	**d.f.**	***F***	***p***	S3
	3 mo	3–28	0.91	0.45	

Birth rates were not measured at month 0 because births were quantified as the number of newly emerged adults since the previous survey (equation (2.1)), and mortality is not reported at month 0 because there was near-zero mortality across all populations at this timepoint. Significant effects (*p* < 0.05) are shown in bold. Significant contrasts from Tukey-adjusted pairwise comparisons are listed here for intrinsic growth rates and carrying capacity, and are shown in figure 1 for all other responses.

The lack of controlled experiments that track population responses to heatwaves over multiple generations has hindered our understanding of the demographic mechanisms that underly lasting responses (but see [15,47]). The consistency and persistence of the suppression in population sizes through time that we observed prompt the question of what caused heatwaves to depress populations in such a sustained manner (figure 1A). Because we allowed our beetles to experience continuous overlapping generations, such that all beetles that were initially added to the experiment and exposed to heatwaves remained in it, the observed reduction in population sizes across our experiment could be the result of negative impacts on the original heatwave-exposed cohort, effects on their descendants across multiple generations, or both. We found that for fecundity and birth rates, the negative effects of heatwaves occurred only early in the experiment, within 1 month of the heatwaves ending (table 1, figure 1B; supplementary material, figure S1). This indicates that adult beetles exposed to heatwaves experienced temporarily reduced reproductive capacity, which is a commonly documented response [16]. At month 3, there was an increase in birth rates in 15-day heatwave populations (figure 1B), likely resulting from populations initially under-shooting their carrying capacity (figure 1A), but this effect was temporary. In contrast to the short-term effects of heatwaves on fecundity and birth rates, the negative effect of heatwaves on mortality rates appeared only later in the experiment, at 5 and 7 months post-heatwave (table 1, figure 1C). Notably, the natural lifespan of our focal species is approximately 6 months [31], and so the increased mortality rates at 5 months could be the result of reduced lifespans of heatwave-exposed beetles, potentially in addition to increased mortality rates of their descendants [28,48]. Overall, the temporal separation between the effects of heatwaves on birth rates and mortality rates meant that when the negative effects of our heatwaves on birth rates had ended (3 months and beyond), the effects on mortality began to appear (5 and 7 months), which created a consistent depression of carrying capacity across the 7-month study (figure 1A). This finding contributes to a growing body of work demonstrating how the effects of environmental change on individuals can scale up to cause more delayed responses at the population level [49].

Further research in other systems is needed to determine how broadly our findings apply. For example, flour beetle life history is characterized by frequent dispersal, colonization and population expansion [26,35], and so populations of *T. castaneum* are unlikely to be at a stable equilibrium for long periods of time. Accordingly, we imposed our heatwaves when our populations were well below carrying capacity, and our heatwaves thus impacted both the growth phase and the carrying capacity of our populations (figure 1A). As such, our findings are most relevant to cases in which heatwaves affect population or communities out of equilibrium, such as including those that experience frequent dispersal, frequent disturbance, transient dynamics, seasonal or shifting climates, or other anthropogenic stressors [50,51]. In addition, the duration and intensity of our heatwaves were relatively modest compared with more extreme events that are becoming increasingly common [40]. Likewise, our study organisms are more tolerant of high temperatures than organisms with temperature-sensitive physiology, such as marine organisms [39]. Because of this, the more subtle effects on population dynamics that we observed here could be overshadowed by high levels of direct heatwave-induced mortality in more heat-sensitive systems or those exposed to longer and more intense heatwaves. And finally, while body size and sex ratios can both mediate population dynamics [52,53], and both can be affected by temperature [54,55], we found no effects of heatwaves on either measure (supplementary material, figures S2 and S3). However, because these traits were only each measured at a single timepoint, we may have missed transient responses, and there are many other traits known to respond strongly to heatwaves that we did not measure, such as egg and larval survival [30] and male fertility [16,28], that could also contribute to the effects of heatwaves on population dynamics in other systems.

Extreme heat events are increasing with climate change, and our study informs the management and conservation of populations faced with an increasing frequency and duration of heatwaves in two important ways. First, our finding that longer heatwaves cause more severe impacts on insect populations indicates that populations in regions predicted to experience prolonged heatwaves (e.g. 10 or more days) could be at increased risk. Second, our finding of longer term responses (reduced carrying capacity) and responses that only appeared several generations after the heatwaves concluded (increased mortality rates) indicates that monitoring only short-term responses to heatwaves may not be sufficient to detect the full effects of temperature extremes on ecological systems. Overall, this research highlights the lasting impacts that heatwaves can have on biological communities and the need for experiments that disentangle the mechanisms underlying these effects.

## Supplementary Material



## Data Availability

All data and code used in this article are archived on Zenodo [46]. Supplementary material is available online [56].
