# Supplementary material for: Persistent effects of experimental heatwave duration on population dynamics
Source: Biol Lett. 2026 Oct 21;22(10):20260444. doi: 10.1098/rsbl.2026.0444 (PMC13682937; doi:10.1098/rsbl.2026.0444)
Supplement: Supplementary file 1 [file rsbl.2026.0444.zip › rsbl.2026.0444/rsbl20260444_si_001.docx]

# Supplementary Material for:

# “Persistent effects of experimental heatwave duration on population dynamics”

# by Emma B. Gruntz and Tess N. Grainger

*Gompertz model description*

To analyze the effect of heatwave duration on population dynamics, we fit a Gompertz population growth model to our population size data. The Gompertz model can be written as $\log\left( \frac{N_{t}}{N_{t-1}} \right)=a+(b-1){log(N}_{t-1})$, where $N_{t}$ is beetle population size at time *t*, *a* is the intrinsic growth rate, and *b* is the strength of density dependence (equation 1 in Ives *et al.* (2003)). We compared this Gompertz model to a Ricker model using used Akaike's Information Criterion (AIC), and because Gompertz had a lower AIC (Gompertz -145.0, Ricker 19.0), we used the Gompertz model in subsequent analyses.

On a log scale, the Gompertz model is the simple linear difference equation $x_{t}=a+bx_{t-1}$, where $x_{t}$ is the natural log of population size at time *t*, $a$ is the population’s intrinsic growth rate, $b$ is the strength of density dependence (equation 2 in Ives *et al.* (2003)). The natural log of carrying capacity can then be calculated as ${\log(K) =x}_{\infty}=a/(1-b)$ (equation 3 in Ives *et al.* (2003)). The intercept in this model corresponds to parameter *a* (intrinsic growth rate) and the slope corresponds to parameter $b$ (the strength of density dependence). We extracted the estimate of *a* and *b* for each population, calculated *K* using the equation above, and used these estimates of *a* and *K* in our analysis (see methods in main text).

**Figure S1.** **Effect of heatwave duration on *T. castaneum* fecundity at (a) 1 month after heatwave exposure and (c) 3 months after heatwave exposure.** See Fig. 1b inset for fecundity results at 0 month after heatwave exposure. Heatwaves were 0 days (control), 5 days, 10 days or 15 days. Heatwaves were set at a constant 38°C, while the control treatment experienced a constant 30°C and no heatwave. Solid points represent the mean population size for each heatwave condition, faded points represent the mean of 3 individuals measured in each population, and error bars show the standard error from the mean.

**Figure S2.** **Effect of heatwave duration on *T. castaneum* body size** **measured 3 months after heatwave exposure.** Heatwaves were 0 days (control), 5 days, 10 days or 15 days. Heatwaves were set at a constant 38°C, while the control treatment experienced a constant 30°C and no heatwave. Solid points represent the treatment means, faded points represent the mean of 5 individuals measured in each population, and error bars show the standard error from the mean.

**Figure S3.** **Effect of heatwave duration on *T. castaneum* sex ratio** **(proportion female), measured 3 months after heatwave exposure.** Heatwaves were 0 days (control), 5 days, 10 days or 15 days. Heatwaves were set at a constant 38°C, while the control treatment experienced a constant 30°C and no heatwave. Solid points represent the treatment means, and faded points show data from each population. Error bars show the standard error from the mean.

**References**

Ives, A.R., Dennis, B., Cottingham, K.L. & Carpenter, S.R. (2003). Estimating community stability and ecological interactions from time‐series data. *Ecol. Monogr.*, 73, 301–330.
